# Augmentation of full-thickness rotator cuff tears with a bioinductive collagen implant does not reduce retear rates – a propensity matched cohort study

**DOI:** 10.1186/s12891-025-09199-2

**Published:** 2025-09-23

**Authors:** Peter Rab, Igor J. Shirinskiy, Michael Kimmeyer, Arno A. Macken, Andrea G. Calamita, Antonio G. Colombini, Geert Alexander Buijze, Thibault Lafosse

**Affiliations:** 1 Alps Surgery Institute, Clinique Générale Annecy, Annecy, France; 2 Department of Sports Orthopaedics, Technical University of Munich, Ismaninger Strasse 22, Munich, 81675 Germany; 3 Shoulder and Elbow Unit, Department of Orthopedic Surgery, OLVG, Amsterdam, The Netherlands; 4 Department of Orthopaedic and Trauma Surgery, University Medical Centre Mannheim, Mannheim, Germany; 5 Department of Orthopaedic Surgery and Sports Medicine, Rotterdam, the Netherlands; 6 A.O.U. Careggi CTO, University of Florence, Florence, Italy; 7 Department of Orthopaedic Surgery, Ospedale Montecchi di Suzzara, Mantova, Italy

**Keywords:** Regeneten, Patch, Tendon augmentation, Supraspinatus, Rotator cuff failure, Sugaya classification, Arthroscopic surgery, Propensity score

## Abstract

**Purpose:**

To compare the clinical and radiographic outcomes after full-thickness RC repair with and without performing augmentation with a bioinductive collagen implant (BCI).

**Materials and methods:**

Consecutive patients who underwent primary repair of a full-thickness supraspinatus tear between 05/2021 and 11/2023 were retrospectively identified. Patients at elevated risk for retear were defined by biological, radiographic, and intraoperative risk factors. Those who underwent repair with or without concomitant augmentation using a BCI and who had both clinical and radiographic follow-up at 1 year postoperatively were matched in a 1:1 ratio according to age, sex, body mass index, tear size, smoking status, diabetes, and American Society of Anesthesiologists physical status classification. Range of motion (ROM) as well as patient-reported outcome measures (Auto-Constant-Score (CS), American Shoulder and Elbow Surgeons (ASES) Score, Subjective Shoulder Value (SSV), and Visual Analog Scale (VAS) for pain) were recorded. Magnetic resonance imaging performed at 1 year postoperatively was analyzed and the presence of retear was recorded.

**Results:**

In total, 149 patients with a radiographic and clinical follow-up at 1 year postoperatively were identified. Of these, 23 patients with BCI augmentation were matched to 23 patients without placement of BCI (48% female, 59.2 ± 8.4 years at surgery). A retear occurred in 5 patients (21.7%) in the BCI augmentation group and in 3 patients (13.0%) in the control group (*p* = 0.72). No significant difference was reported regarding the CS (77 [71–83] vs. 76 [63–81], *p* = 0.5), ASES Score (92 [82–98] vs. 90 [84–95], *p* = 0.8), SSV (90 [80–100] vs. 90 [88–95], *p* = 0.9), VAS for pain (*p* = 0.74), or ROM between the groups.

**Conclusion:**

In this retrospective matched cohort of patients at elevated risk for retear, augmentation of full-thickness RC repair with a BCI was not associated with a reduced retear rate. Moreover, no significant differences regarding clinical and functional outcome were found between the two groups.

**Level of evidence:**

III – Retrospective case series with a matched control group.

**Supplementary Information:**

The online version contains supplementary material available at 10.1186/s12891-025-09199-2.

## Introduction

Rotator cuff (RC) tears are a leading cause of shoulder-related disability, with a substantial socioeconomic burden resulting in a considerable impact on the working population [1]. While the initial treatment typically includes conservative management, particularly in the context of partial-thickness tears, surgical intervention is proposed for patients with persistent symptoms under non-surgical treatment [2–4]. Despite considerable advances in surgical techniques and materials, a retear after RC repair remains a major concern with reported retear rates of up to 94% for massive RC tears [5–9]. Tear size, muscle atrophy, retraction and fatty infiltration have been reported to strongly influence the risk for retear, and high-risk patients have become a key concern for surgeons [10–14]. In addition, patient-specific biological factors such as age, diabetes, smoking and obesity have been described to increase the risk of failed healing after RC repair [15–20].

Substantial efforts have been made to improve tendon healing after RC repair and a variety of patches and collagen scaffolds have been developed using animal, human or synthetic materials [21]. While earlier xenograft patch augmentations yielded inconsistent outcomes, recent advances using bovine collagen scaffolds have demonstrated promising results. These scaffolds may enhance tendon healing by promoting collagen formation and increasing collagen fiber density, thereby addressing the adverse biological environment of degenerative rotator cuff tears [21–31]. The Regeneten bioinductive bovine collagen implant (BCI) (Smith & Nephew, Andover, MA) has been associated with favorable outcomes and low retear rates, initially introduced for the treatment of partial-thickness tears and subsequently expanded to augment full-thickness tears [23, 26–29]. However, there is a paucity of comparative or case-control studies in the literature. Therefore, the aim of this study was to compare the clinical and radiographic outcomes, including retear rates, of patients at elevated risk for retear undergoing full-thickness RC repair with and without augmentation with a BCI. The hypothesis was that BCI augmentation in patients at elevated risk for retear would be associated with a lower retear rate and superior patient-reported outcomes compared to standard RC repair.

## Materials and methods

This single-center, retrospective comparative study was approved by the institutional review board and conducted in accordance with the Declaration of Helsinki. A retrospective search was conducted to identify all consecutive patients who underwent primary arthroscopic supraspinatus (SSP) repair between May 2021 and November 2023. Exclusion criteria were revision surgery for previously failed RC repair, isolated subscapularis tendon (SSC) repair, only partially repaired SSP tears, and concomitant procedures other than SSC repair or LHBT tenodesis/tenotomy. For the matching process, only patients with clinical and radiologic follow-up with MRI at 1 year postoperatively were selected.

### Preoperative assessment and surgical planning

Preoperatively, all patients underwent standardized anteroposterior and y-view radiographs and Magnetic Resonance Imaging (MRI) or Computer Tomographic imaging with intraarticular contrast fluid injection. Retraction of SSP according to Patte, the extent of fatty infiltration, as classified by Goutallier, and muscle atrophy, as defined by Thomazeau, were assessed [32–34]. Patients with fatty infiltration > 2 or atrophy > 2 were excluded from undergoing RC repair, as they underwent alternative operative or nonoperative treatments. The decision to use a BCI was based on biological, radiologic and intraoperative factors. Regarding biological patient-specific risk factors, an American Society of Anesthesiologists physical status classification (ASA score) [35, 36] greater than 1, a body mass index (BMI) greater than 28, the presence of diabetes mellitus, rheumatoid arthritis, or active smoking were considered risk factors for retear [15, 37, 38]. In addition, an acromiohumeral distance of less than 7 mm and an increased critical shoulder angle (CSA) greater than 34° were considered risk factors for RC retear [15, 38]. Intraoperatively, tendon fraying and calcification were noted as signs of poor tendon quality and a higher risk for retear [39, 40]. The use of BCI was indicated if four or more risk factors were present. Due to the introductory phase of the BCI and resulting intermittent logistical unavailability, not every patient received a scaffold, even if the number of risk factors indicated eligibility. The intermittent availability of the BCI was not associated with any patient characteristics apart from time of presentation at the outpatient clinic and was therefore considered a random factor, which made a retrospective matched analysis feasible.

### Surgical technique

The surgical procedure was performed by two fellowship-trained shoulder surgeons (T.L. and G.A.B.) in the beach chair position and under general anesthesia. Single- or double-row arthroscopic rotator cuff repair was performed based on tear size, with single-row repair used for tears up to 1.5 cm, using non-metallic suture anchors with two sutures. In the event of BCI (Regeneten; Smith & Nephew, Andover, MA) placement, the size (medium or large) was selected based on the dimensions of the RC tear and positioned on the repaired SSP tendon. BCI placement was performed as previously described [41]. Soft-tissue fixation in the SSP was performed using six to eight polydioxanone anchors placed via a cannula, while fixation in the greater tuberosity was achieved using two polyether ether ketone anchors. The repair of the SSC was performed in cases of Lafosse stage II or higher, using either a single-row or double-row technique [42]. LHBT tenodesis and subacromial decompression were performed in all patients. In patients with osteoarthritis of the acromioclavicular (AC) joint, resection of the lateral clavicle combined with the articular face of the acromion was performed. Postoperative immobilization in 30° abduction was maintained for a six-week period, while pain-free mobilization and pendulum exercises were allowed. Active rehabilitation started at six weeks postoperatively, with resistance exercises introduced at three months.

### Matching process

Matching was performed using R version 4.2.1 (R Foundation for Statistical Computing, Vienna, Austria) and the *MatchIt* package [43]. A propensity score was calculated with the following matching criteria of equal weights: age, sex, BMI, tear size (on an ordinal a scale from 1 to 3: 1, small tear < 1 cm; 2, medium or large tear between 2 and 5 cm; 3, massive tear > 5 cm) [44], smoking (yes/no), diabetes mellitus type 1 or 2 (yes/no), and ASA score (1 to 4). According to the matching criteria, each patient who received an intraoperative BCI was matched to one patient without BCI placement if they met four or more of the aforementioned biological, radiographic, or intraoperative risk factors for re-tear. A genetic matching algorithm, an extension of propensity score matching with iterative verification of balance, was performed to reduce conditional bias, and to counteract disadvantages of isolated propensity score matching [45–47]. Cases with missing data were excluded from the analysis and the matching process. The standardized differences for all covariates were calculated both before and after matching, with a threshold of 10% or more deemed indicative of imbalance [48].

### Outcome assessment

Clinical examination was performed, and patient-reported outcome measures (PROM) were obtained pre-operatively and after a minimum of 12 months postoperatively. The PROMs included the Auto-Constant-Score (CS), a Visual Analog Scale (VAS) for pain, Subjective Shoulder Value (SSV), Disabilities of the Arm, Shoulder and Hand score (DASH), and American Shoulder and Elbow Surgeons score (ASES). Further, tendon healing of the SSP was analyzed in T2 weighted images on MRI according to Sugaya et al. [49], with all scans performed on two identical 3-Tesla machines from the same manufacturer. All MRIs were assessed independently by two authors (P.R. and I.J.S.), with all disagreements resolved by consensus discussion with the senior author (T.L.). Postoperative complications, defined as any adverse event to the same shoulder resulting in temporary or permanent complaints or decreased function, were recorded.

### Statistical analysis

Statistical analysis was performed according to a pre-defined plan using RStudio (RStudio Public Benefit Corporation, Boston, USA) and R version 4.2.1 (R Foundation for Statistical Computing, Vienna, Austria). The Shapiro-Wilk test and histograms were used to assess normality in the distribution of the data. Comparison of scores at different time points was performed using paired t-test for normally distributed variables and Wilcoxon signed rank test for skewed variables. Categorical variables were analyzed using the Fisher exact test. Comparison between the two groups after matching were performed using paired t-tests, Wilcoxon test, and McNemar’s Test, respectively. A sample size calculation was performed to detect a significant difference in the ASES score between the two groups using G*Power (G*Power 3.1, Düsseldorf, Germany) [50]. Based on previous studies [51, 52], the effect size was set to 0.5, with two degrees of freedom, and the significance level was set to 0.05. A minimum of 34 observations was determined to achieve a power of 0.8. The significance level was set at *p* = 0.05, and all tests were two-tailed.

## Results

**Fig. 1:**
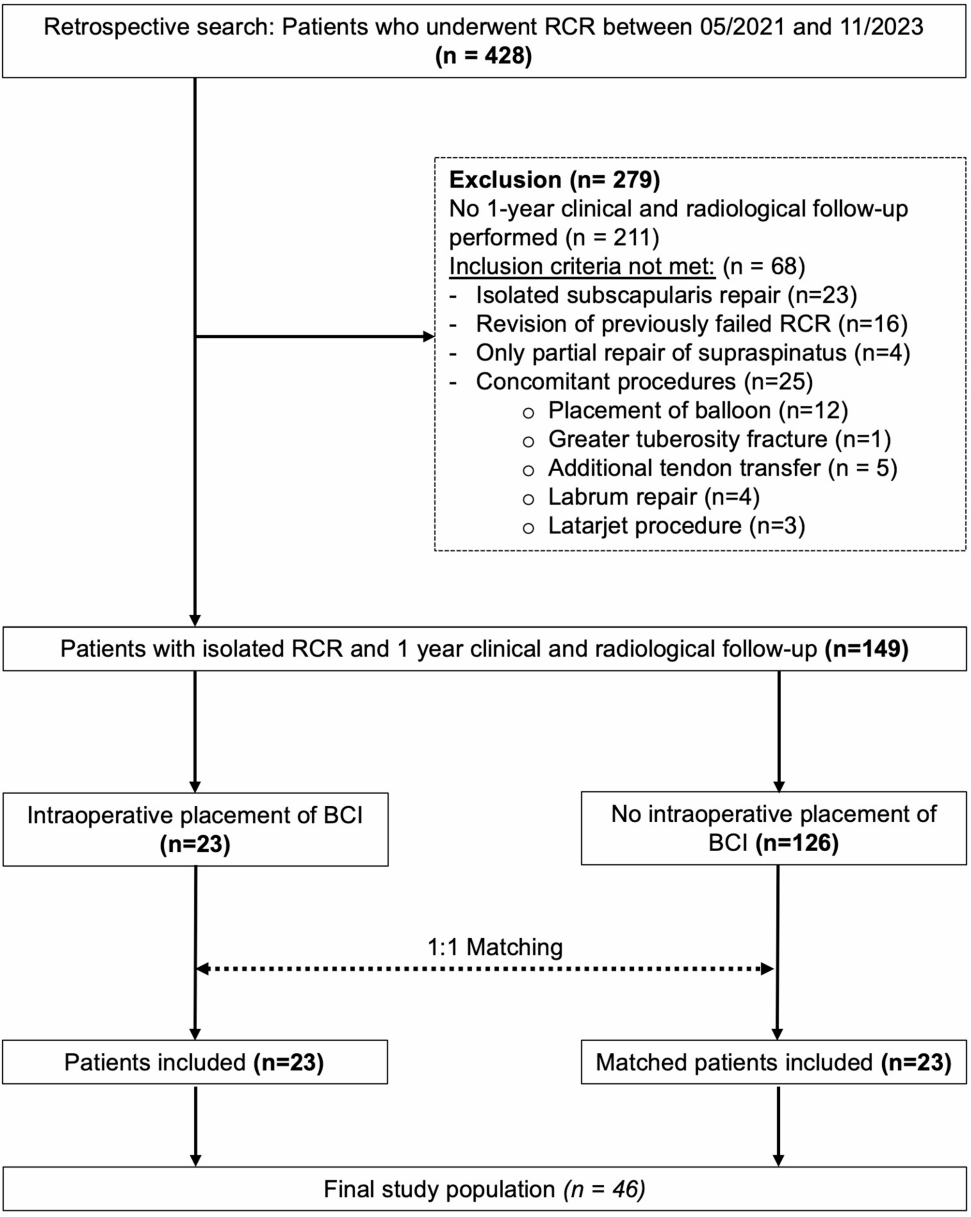
Flowchart of Patient inclusion and matching. BCI, bioinductive bovine collagen scaffold; RCR, rotator cuff repair

In total, 428 patients were retrospectively identified and 23 procedures with intraoperative placement of BCI and 126 procedures without placement of BCI were included (Fig. 1). The 23 patients with BCI were matched to 23 patients without BCI. In the matched cohort, 22 patients (48%) were female, and the mean age was 59.2 ± 8.4 years at time of surgery. For all matching covariates, a standardized difference of less than 4% was observed, indicating an adequate match (Supplementary Tables 1 and 2, Supplementary Fig. 1). No significant difference was found regarding the matching parameters as well as further risk factors after 1:1 matching (Table 1). A concomitant SSC repair was indicated and performed significantly more often in patients who did not receive a BCI (*p* = 0.038).

**Table 1:** Patient cohort demographics, preoperative clinical and radiographic assessment, and surgery characteristics. Significant *p*-values are bolded. AHD, acromiohumeral distance; ASES, American shoulder and elbow surgeons; CSA, critical shoulder angle; IQR, interquartile range; LHBT, long head of the biceps tendon; VAS, visual analog scale

	Treated (*n*=23)	Control (*n*=23)	*p*-value
Patient demographics and anamnestic factors			
Age at surgery, mean ± standard deviation	59.2 ± 9.4	59.6 ± 7.7	0.96
Female, *n* (%)	11 (48)	10 (43)	0.15
BMI, median [IQR]	25.7 [22.5 – 30.1]	24.7 [22.9– 30.7]	0.89
Smoking, *n* (%)	5 (22)	5 (22)	1
Diabetes Mellitus, *n* (%)	3 (13)	3 (13)	–
ASA-Score	I: 16 (70) II: 6 (48) III: 1 (4) IV: 0	I: 15 (65) II: 8 (39) III: 0 IV: 0	0.75
Rheumatoid arthritis,* n* (%)	0	0	–
Previous corticosteroid injections, *n* (%)	15 (65)	17 (74)	0.52
Traumatic tear, *n* (%)	13 (56)	17 (74)	0.52
Dominant side operated, *n* (%)	13 (56)	17 (74)	0.21
Clinical and functional parameters			
Anterior elevation, median [IQR]	160 [120 - 170]	160 [115 - 179]	0.93
External rotation, median [IQR]	45 [38 - 53]	45 [45 - 60]	0.23
Subjective Shoulder Value, median [IQR]	50 [48 - 65]	50 [40 - 60]	0.70
VAS for pain, median [IQR]	5 [4 - 7]	6 [5 - 6]	0.37
Constant-Score, median [IQR]	42 [23 - 53]	36 [24 - 47]	0.64
ASES score, median [IQR]	49 [41 - 59]	49 [41- 60]	0.31
Radiologic parameters			
Calcification of supraspinatus, *n* (%)	7 (30)	3 (13)	0.15
CSA >34°, *n* (%)	12 (40)	15 (65)	0.37
AHD < 7mm, *n* (%)	2 (9)	1 (4)	0.55
Fatty infiltration (Goutallier ≤ 3), *n* (%)	0	0	–
Positive tangent sign, *n* (%)	0	0	–
Intraoperative findings			
Tear size, *n* (%)	Small 4 (17) Medium/Large 8 (35) Massive 11 (48)	Small 3 (13) Medium/Large 10 (43) Massive 10 (43)	0.85
Supraspinatus tear retraction (Patte), *n* (%)	I 7 (30) II 7 (30) III 9 (39)	I 4 (17) II 11 (48) III 8 (35)	0.41
Concomitant subscapularis repair, *n* (%)	9 (39)	16 (67)	**0.038**
Concomitant LHBT tenodesis, *n* (%)	23 (100)	23 (100)	**–**
Concomitant acromioplasty, *n* (%)	23 (100)	23 (100)	–
Number of anchors placed, median [IQR]	3 [2 - 4]	4 [2 - 4]	0.71
Double row repair, *n* (%)	16 (70)	16 (70)	–
Surgery time, minutes [IQR]	90 [79 - 116]	74 [61 - 88]	**0.002**

At 12 months postoperatively, no significant difference was reported in the CS (77 [71–83] vs. 76 [63–81], *p* = 0.5), ASES score (92 [82–98] vs. 90 [84–95], *p* = 0.8), SSV (90 [80–100] vs. 90 [88–95], *p* = 0.9), or VAS for pain (1.0 [0–2.4] vs. 1.0 [0.7–2.0], *p* = 0.74). Moreover, no significant difference in ROM was observed between the two groups (Table 2). A SSP retear (Sugaya 4 or 5) was observed in 5 patients (21.7%) in the BCI augmentation group and in 3 patients (13.0%) in the control group, with no significant difference between the groups (*p* = 0.72). The overall retear rate was 17.4% (*n* = 8). In the group that received a BCI, 6 complications (30%) including RC retear were observed (Table 2). Of these, one patient underwent a subpectoral LHBT tenodesis 12 months after the index procedure for symptomatic popeye sign. In the control group a total of 6 complications (26%) were reported, no patient underwent revision surgery. No significant difference in complication rates was observed between the two groups (*p* = 1). No retear of the SSC was observed in any group.

**Table 2:** Postoperative outcome parameters. ASES, American shoulder and elbow surgeons; IQR, interquartile range; VAS, visual analog scale

	Treated (*n*=23)	Control (*n*=23)	*p*-value
Follow-up time, months [IQR]	12.2 [11.5 – 12.9]	12.5 [11.7 – 13.0]	0.43
Clinical and functional outcome			
Anterior elevation, median [IQR]	170 [165 - 180]	180 [170 - 180]	0.39
Abduction, median [IQR]	170 [160 - 180]	180 [160 - 180]	0.34
External rotation, median [IQR]	50 [45 - 60]	45 [45 - 50]	0.07
Internal rotation, median [IQR]	T10 [T10 - T8]	T10 [T12 - T8]	0.52
Subjective Shoulder Value, median [IQR]	90 [80 - 100]	90 [88 - 95]	0.91
VAS for pain, median [IQR]	1.0 [0 – 2.4]	1.0 [0.7 – 2.0]	0.74
Constant-Score, median [IQR]	77 [71 - 83]	76 [63 - 81]	0.53
ASES, median [IQR]	92 [82 - 98]	90 [84 – 95]	0.81
Radiographic outcome			
Retear, *n* (%)	5 (22)	3 (13)	0.72
Tendon healing (Sugaya Score), *n* (%)			0.8
SUGAYA 1/2	15 (65)	16 (70)	
SUGAYA 3	3 (13)	4 (17)	
SUGAYA 4/5	5 (22)	3 (13)	
Fatty infiltration Goutallier ≥ 3,*n* (%)	1 (4)	1 (4)	–
Complications, *n* (%)	7 (30%)	6 (26%)	1
Type of complications	Retear (*n* = 5) Symptomatic Popeye (*n* = 1) Stiffness (*n* = 1)	Retear (*n* = 3) Symptomatic Popeye (*n* = 1) Adhesive capsulitis (*n* = 1) CRPS (*n* = 1)	

## Discussion

This study aimed to compare the clinical and radiographic outcomes of patients who underwent primary arthroscopic RC repair with biological augmentation using a BCI with those of patients who did not receive biological augmentation, in a matched cohort of patients at elevated risk for retear. No significant differences were observed between the two groups regarding baseline characteristics or risk factors for retear, However SSC repair was performed significantly more often in the control group. At 1 year postoperatively, no significant differences were observed regarding retear rate, complication rate, or clinical and functional outcome.

Despite substantial advancements in surgical techniques for RC repair recent decades, the occurrence of retear remains a major concern, with considerable socioeconomic implications [1, 6, 8, 53]. In this study, notably higher retear rates were observed overall (17.4%) as well as in both groups compared to retear rates previously reported in literature on the general population who underwent RC repair [7, 14, 54]. Moreover, Warren et al. reported an overall retear rate of 8% for patients with augmentation using a BCI for full-thickness RC tears in a recent meta-analysis [55]. However, this discrepancy may be attributed to the inclusion of patients at elevated risk of retear due to biologic and radiographic factors, as well as a large proportion with massive RC tears, which likely contributed to the higher observed retear rate [56]. As a result, it is challenging to extrapolate findings from a cohort at elevated risk for retear to other studies and the general population.

Although several authors evaluated the effects of BCI augmentation in full-thickness tears, few studies to date have assessed the outcomes of BCI augmentation in RC repair in comparison with a control group. In accordance with the findings of the present study, Zhang et al. reported no difference in patient-reported outcomes and retear rates in large to massive RC tears [46]. However, matching was performed by tear size only, and clinical and radiographic analysis was performed at 6 months postoperatively, which limits comparability. In a propensity-score matched analysis, Haft et al. observed no superiority in patient-reported outcomes between patients with and without augmentation with a BCI, in patients with partial or full-thickness RC tears, concordant with the results of the present study [57]. However, a higher reoperation rate due to stiffness and inflammation was reported in patients who received a bovine collagen scaffold, which was not observed in the present cohort. Notably, no postoperative radiographic analysis was performed, and retear rates could not be reported.

To date, only two randomized controlled trials (RCT) have evaluated the effect of BCI augmentation of full-thickness tears. Camacho Chacón et al. compared a double row SSP repair to only debridement and augmentation with a BCI in patients with a small to medium full-thickness RC tear in an RCT [58]. Contrary to the findings of the present study, the group that received a BCI demonstrated significantly higher CS and ASES scores, as well as significantly lower pain levels. Furthermore, a 100% healing rate was observed in the group receiving a BCI at 12 and 24 months follow-up. The retear rate in the control group was not reported [58]. However, the comparability with the present study is limited by the inclusion of only small-to-medium RC tears with an intact rotator cable and the comparison of repair versus BCI augmentation alone. Further, Ruiz Ibán et al. reported a significantly reduced retear rate (8.3% vs. 25.8%) in full-thickness medium to large posterosuperior RC tears in a recent RCT [59]. Notably, no significant difference was observed in functional outcome or pain levels, which is consistent with the findings of the present study. Although the patient cohort was similar with respect to risk factors for retear in comparison to the present study, no patients with repair of the SSC were included. A potential further rationale for the different result regarding retear rates may be due to reduced selection bias and surgeon preference as a result of the randomized controlled trial design and larger sample size. In conclusion, the limited number RCTs evaluating BCI augmentation has primarily focused on the general population undergoing RC repair. In contrast, the present study specifically targeted a patient cohort at elevated risk for retear, underscoring the necessity for further research to assess the benefits of BCI augmentation in this subgroup.

In this study, the hypothesis that BCI augmentation in patients at elevated risk for retear would be associated with lower retear rates and superior patient-reported outcomes could not be confirmed. Moreover, while no significant difference in retear rates was observed, the numerical retear rate was higher in the BCI group (22% vs. 13%). Although this finding may be confounded by cohort heterogeneity, such as the inclusion of both single- and double-row repairs and concomitant SSC repair, possible explanations include tendon overload or impaired tendon-bone integration caused by the BCI, warranting further investigation. Considering the findings of the present study and the inconsistent results reported in the literature, it remains challenging to propose a recommendation regarding which patient may benefit optimally from biologic augmentation. Despite the selection of patients at elevated risk for retear and matching being predominantly performed according to well-established biologic risk factors for retear, no superiority in outcomes was demonstrated in this study. Consequently, a general recommendation to biologically augment RC repair primarily according to biologic risk factors cannot be made. Although a previous decision analytic model suggested a cost-effectiveness ratio in favor for the use of the BCI, a significant and notable reduction in retear rate must be consistently demonstrated in the literature as it is the underlying premise for a potential economic benefit [60]. This highlights the necessity for further research comparing the outcomes of BCI augmentation to standard RC repair in larger, well-designed RCTs, particularly in the patient population at elevated risk for re-tear.

To address the inconsistent findings in literature, a distinction between tear sizes and morphology may prove beneficial. In small full-thickness SSP tears, the findings of Camacho Chacón et al. suggest a beneficial effect of using a BCI, and a meta-analysis of Warren et al. observed a low mean retear rate of 1.1% in partial-thickness tears [55]. Conversely, the findings of Zhang et al. suggest a less reliable and consistent benefit of BCI in large to massive RC tears, as no superiority in outcomes could be demonstrated [61]. In the broader context of revision RC repair, two studies found no difference in functional outcomes or retear rates, as demonstrated by Tisherman et al. and Ting et al. [62, 63]. Further research with specifically selected tear sizes or larger cohorts with the possibility of regression analysis may be able to assess the influence of tear size on biological augmentation with a BCI.

## Limitations

This study has several limitations. First, the sample size of 46 patients as well as the selection of patients at elevated risk for retear may limit the external applicability of the results and may have been underpowered to detect a significant difference in retear rates. Moreover, while matched cohort studies mitigate selection bias inherent to retrospective analyses and improve comparability between groups, some degree of residual selection bias may persist, influenced by factors such as intraoperative decision-making. Although the time factor in this study was considered near-random due to the intermittent availability of the BCI, a residual bias may persist and may only be eliminated by conducting a randomized controlled trial. While the genetic matching algorithm employed in this study improves covariate balance and reduces conditional bias, propensity score-based matching across multiple covariates may still introduce some bias*.* Furthermore, while the semiquantative classification of RC tear sizes used in the present study facilitates the matching process and calculation of a propensity score, it may limit comparability and lead to a lower degree of accuracy. In addition, although the Sugaya classification is widely used to assess RC healing, there are limitations in its semiquantitative three-dimensional assessment, sensitivity, and predictive value, as well as in distinguishing between a Sugaya 3 and a Sugaya 4 retear, and in the clinical implications of a Sugaya 3 tendon healing [64, 65]. Lastly, the heterogeneity of the study cohort with different degrees of tear size and risk factors, although representative of clinical practice, further limits interpretation and extrapolation of the results to the general population. As such, surgical variables, including the number of anchors and the inclusion of single- and double row repair as well as concomitant procedures, such as LHBT tenotomy or tenodesis, SSC repair, infraspinatus repair, acromioplasty, and AC joint resection, may have influenced clinical outcomes and introduced further bias.

## Conclusion

In this retrospective matched cohort with respect to risk factors for retear with 1 year follow-up, BCI augmentation of full-thickness RC repair in patients at elevated risk for retear did not result in a reduced retear rate. Further, no significant differences regarding clinical and functional outcome were reported between the two groups.

## Supplementary Information

Supplementary Material 1

## Data Availability

All data generated or analyzed during this study are included in this published article.

## Abbreviations

AC: Acromioclavicular
ASA: American Society of Anesthesiologists
ASES: American Shoulder and Elbow Surgeons score
BCI: Bioinductive bovine collagen implant
BMI: Body Mass Index
CSA: Critical Shoulder Angle
CS: Constant-Score
DASH: Disabilities of the Arm, Shoulder, and Hand score
LHBT: Long Head of the Biceps Tendon
MRI: Magnetic Resonance Imaging
PROM: Patient-Reported Outcome Measures
RC: Rotator Cuff
ROM: Range of Motion
SSC: Subscapularis
SSP: Supraspinatus
SSV: Subjective Shoulder Value
VAS: Visual Analog Scale
