# Supplementary material for: Augmentation of full-thickness rotator cuff tears with a bioinductive collagen implant does not reduce retear rates – a propensity matched cohort study
Source: BMC Musculoskelet Disord. 2025 Sep 23;26:855. doi: 10.1186/s12891-025-09199-2 (PMC12455770; doi:10.1186/s12891-025-09199-2)
Supplement: Supplementary file 1 — Supplementary Material 1 [file 12891_2025_9199_MOESM1_ESM.docx]

Supplementary Table 1: Matching covariates in the treated population and the matched and not matched control cohort. ASA, American Society of Anesthesiologists physical status classification; BMI, Body Mass Index; SD, standard deviation

| Covariate | Treated (n=23) | Matched control (n=23) | | Not matched control (n=103) | *p*-value  (treated vs. matched control) | *p-*value  (treated vs. not matched control) |
| --- | --- | --- | --- | --- | --- | --- |
| Age at surgery, mean ± SD | 59.2 ± 9.4 | 59.6 ± 7.7 | 60.6 ± 9.6 | | 0.96 | 0.72 |
| Female, n (%) | 11 (48) | 10 (43) | 44 (44) | | 0.15 | 0.74 |
| BMI, median [IQR] | 25.7 [22.5 – 30.1] | 24.7 [22.9– 30.7] | 24.1 [22.9 – 28.2] | | 0.89 | 0.91 |
| Smoking, n (%) | 5 (22) | 5 (22) | 13 (13) | | 1 | 0.29 |
| Diabetes Mellitus, n (%) | 3 (13) | 3 (13) | 2 (2) | | – | **0.028** |
| ASA-Score | I 16 (70) II 6 (48)  III 1 (4)  IV 0 | I 15 (65) II 8 (39)  III 0  IV 0 | I 58 (56) II 45 (44)  III 0 (0)  IV 0 | | 0.75 | 0.35 |
| Tear size, n (%) | Small: 4 (17)  Medium/Large: 8 (35)  Massive: 11 (48) | Small: 3 (13)  Medium/Large:10 (43)  Massive: 10 (43) | Small: 29 (28)  Medium/Large: 55 (53)  Massive: 19 (18) | | 0.85 | **0.021** |

Supplementary Table 2: Standardized mean differences overall and for each covariate before and after matching. ASA, American Society of Anesthesiologists physical status classification; BMI, Body Mass Index; St. Mean Diff., Standardized mean difference.

| Covariate | Std. Mean Diff. before matching | Std. Mean Diff. after matching | Improvement |
| --- | --- | --- | --- |
| Overall Distance | 0.5774 | -0.0247 | 0,553 |
| Age at Surgery | -0.0735 | -0.0052 | 0,068 |
| Sex | -0.5565 | 0.0000 | 0,557 |
| ASA | -0.1530 | 0.0000 | 0,153 |
| Tear size | -0.1381 | -0.0147 | 0,123 |
| BMI | 0.0586 | -0.0141 | 0,045 |
| Diabetes Mellitus | 0.2676 | 0.0000 | 0,268 |
| Smoking | 0.1751 | 0.0000 | 0,175 |

Supplementary Figure 1: A, Love Plot of Standardized mean differences before and after genetic propensity matching, B, Balance plot of overall distance before and after genetic propensity score matching. ASA, American Society of Anesthesiologists physical status classification; BMI, Body Mass Index
